# Evaluating a Clinical Decision Support Tool for Cancer Risk Assessment in Primary Care: Simulation Study of Unintended Weight Loss

**DOI:** 10.2196/79208

**Published:** 2025-12-10

**Authors:** Javiera Martinez-Gutierrez, Sophie Chima, Lucas De Mendonca, Alex Lee, Barbara Hunter, Jo-Anne Manski-Nankervis, Deborah Daly, George Fishman, Kit Huckvale, Fong Seng Lim, Benny Wang, Craig Nelson, Brian Nicholson, Jon Emery

**Affiliations:** 1 Department of Family Medicine, Faculty of Medicine, Pontificia Universidad Catolica de Chile, Santiago, Chile; 2 Department of General Practice and Primary Care, Faculty of Medicine, Dentistry and Health Sciences, The University of Melbourne, Medical Building, Grattan Street, Parkville, Australia, 61 390355511; 3 Collaborative Centre for Genomic Cancer Medicine, The University of Melbourne, Parkville, Australia; 4 Primary Care and Family Medicine Department, Lee Kong Chian School of Medicine, Nanyang Technological University, Singapore, Singapore; 5 Primary Care Collaborative Cancer Clinical Trials Group (PC4), Melbourne, Australia; 6 The Digital Health Validitron, Centre for Digital Transformation of Health, The University of Melbourne, Parkville, Australia; 7 National University Polyclinics, Singapore, Singapore; 8 Singapore Primary Care Cancer Network (SPriNT), Singapore, Singapore; 9 Western Health Chronic Disease Alliance, Western Health Melbourne, Melbourne, Australia; 10 Department of Medicine, Western Health, The University of Melbourne, Melbourne, Australia; 11 Nuffield Department of Primary Care Health Sciences, University of Oxford, Oxford, United Kingdom

**Keywords:** digital health, clinical decision support, cancer prevention, early detection, general practice, primary care

## Abstract

**Background:**

Early cancer detection is crucial, but recognizing the significance of associated symptoms such as unintended weight loss in primary care remains challenging. Clinical decision support systems (CDSSs) can aid cancer detection but face implementation barriers and low uptake in real-world settings. To address these issues, simulation environments offer a controlled setting to study CDSS usage and improve their design for better adoption in clinical practice.

**Objective:**

This study aimed to evaluate a CDSS integrated within general practice electronic health records aimed at identifying patients at risk of undiagnosed cancer.

**Methods:**

The evaluation of a CDSS to identify patients with unintended weight loss was conducted in a simulated primary care environment where general practitioners (GPs) interacted with the CDSS in simulated clinical consultations. There were four possible clinical scenarios based on patient gender and risk of cancer. Data collection included interviews with GPs, cancer survivors (lived-experience community advocates), and patient actors, as well as video analysis of GP–CDSS interactions. Two theoretical frameworks were employed for thematic interpretation of the data.

**Results:**

We recruited 10 GPs and 6 community advocates, conducting 20 simulated consultations with 2 patient actors (2 consultations per GP: 1 high-risk consultation and 1 low-risk consultation). All participants found the CDSS acceptable and unobtrusive. GPs utilized CDSS recommendations in three distinct ways: as a communication aid when discussing follow-up with the patient, as a reminder for differential diagnoses and recommended investigations, and as an aid to diagnostic decision-making without sharing with patients. The CDSS’s impact on patient–doctor communication varied, facilitating and hindering interactions depending on the GP’s communication style.

**Conclusions:**

We developed and evaluated a CDSS for identifying cancer risk in patients with unintended weight loss in a simulated environment, revealing its potential to aid clinical decision-making and communication while highlighting implementation challenges and the need for context-sensitive application.

## Introduction

Cancer remains one of the most significant health burdens in modern society, with early detection playing a crucial role in improving survival rates. However, identifying cancer in primary care settings presents a considerable challenge due to the nonspecific nature of many presenting symptoms [1,2]. Fewer than 50% of patients with cancer initially present to general practice with *red-flag* symptoms (eg, site-specific symptoms such as a breast lump); however, they more commonly present with nonspecific signs and symptoms [3]. For patients with cancer, presenting with nonspecific symptoms can introduce diagnostic complexity, as such symptoms are often present in other, more common diseases.

An important but nonspecific presentation of cancer is unintended weight loss (UWL), which is associated with 10 different types of cancer as well as several other conditions in primary care [4-6]. However, it represents a clinical challenge, especially when occurring as an isolated symptom. A large cohort study showed that patients with UWL have a risk of 3% or more to be diagnosed with cancer [7], which is an international threshold for urgent investigation [8]. Despite being a risk factor for many cancers, clinical recognition as a symptom of cancer is low, and studies have shown that UWL is not easily identified or acted upon in clinical practice [9,10]. Timely investigations of cancer in general practice are vital, with a need to identify patients who are at increased risk of an undiagnosed cancer.

Clinical decision support systems (CDSSs) have been developed to aid in early cancer detection and are generally well-accepted by health care professionals [11]. Briefly, a CDSS utilizes algorithms underpinned by clinical guidelines and academic literature, combining this with patient information to provide guideline or evidence-based recommendations to health care professionals, often at the point of care [12,13]. However, many well-designed, usable, and acceptable CDSSs have low uptake when embedded in real-world clinical practice. A study in the United Kingdom showed that only 17% of general practitioners (GPs) with access to a CDSS for cancer risk in their practice were likely to use it [14]. The real impact of CDSSs in improving clinical care has been described as low to moderate [15].

There are several barriers to the implementation of CDSSs in real-world settings. A systematic review on the use of a CDSS for early cancer detection in primary care described mistrust in the recommendations, lack of time, and the CDSS interfering with the clinical workflow as just some of these barriers. [11]. To address these challenges and gain insights into the practical application of CDSSs without exposing patients to potential risks, simulation clinical environments or laboratories offer a cost-effective alternative to piloting prototype CDSSs in real-life scenarios, particularly for less common clinical presentations. Simulation laboratories to test digital interventions provide an opportunity to observe interactions between health care providers and the CDSS, allowing researchers to study how clinicians actually use the tool in a controlled environment. This approach enables the collection of valuable data not only on tool usage but allows researchers to observe the interaction and assess workflow, communication, and other factors that influence adoption. They can also facilitate discussions about potential improvements by gaging users’ impressions immediately after the interaction has occurred [16].

In light of these considerations, we sought to observe the use of a CDSS designed to aid in the recognition of UWL as a potential cancer symptom. Our study aimed to assess both GPs’ and patients’ perceptions of such a tool, with the ultimate goal of improving early cancer detection in primary care settings.

## Methods

### The Future Health Today Tool

**Figure 1.**
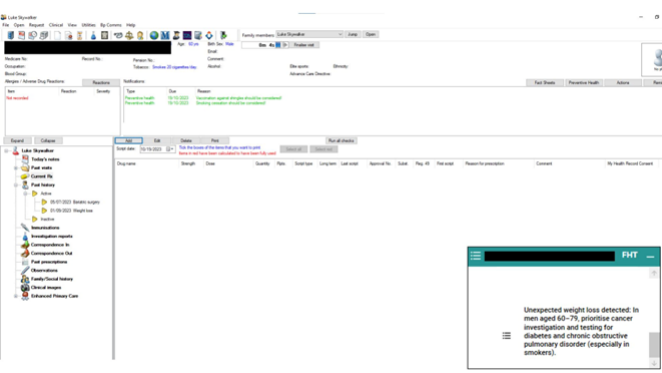
Example of a recommendation for unexpected weight loss as it appeared in the general practice electronic medical record (fictitious patient).

Future Health Today (FHT) is a software that can be integrated with a general practice electronic medical record (EMR) [17]. It consists of two core components: a CDSS point of care prompt and a web-based portal, which includes an audit and recall tool, quality improvement management, and access to education and other resources. The prompt also provides direct access to the reasons for the recommendations and evidence-based information relevant to follow-up. FHT has been co-designed with GPs and consumers, with the purpose of optimizing patient care [18]. Different modules are developed for use in FHT. The FHT UWL module was developed to flag patients with a symptom of UWL recorded in the medical record in the past 6 months. The UWL algorithms take into account age, sex, and any recent abnormal test results, with different recommendations dependent on these factors. An example of a recommendation as it appears in the EMR is shown in Figure 1.

### Simulated Consultations

The study was carried out in the University of Melbourne’s Digital Health Validitron SimLab at the Centre for Digital Transformation of Health [16,19]. The SimLab has a simulated general practice clinic, incorporating a virtual “sandbox” (a virtual machine that is used to run software in a testing environment), which allows for the UWL algorithm to be used on the Best Practice and Medical Director General Practice EMRs in “near to real” scenarios. It is estimated that approximately two-thirds of Australian general practices use Best Practice and one third use Medical Director, with approximately 3.6% of practices using other EMRs [20].

Four hypothetical patient scenarios were designed to align with the UWL module recommendations. These included one female high-risk and one female low-risk scenario and one male high-risk and one male low-risk scenario. Each GP was exposed to one high-risk and one low-risk scenario according to the sex of the actor available. A summary of the scenario characteristics can be found in Table 1. The full patient scenarios can be found in Multimedia Appendix 1. The simulated consultations were performed by actors from the Department of Medical Education at the University of Melbourne, who were experienced in delivering simulated patient (SP) scenarios. The definition of an actor in this scenario was a professional “trained to reproduce the components of real clinical experience” [21].

**Table 1.** Hypothetical patient scenarios for the simulated consultations: overview of patient characteristics.

Sex and risk level	Age (y)	Symptoms	Other risk factors
Female			
High	65	Concerned about UWL^a^, fatigue	New onset diabetes, anemia, and thrombocytosis
Low	60	History of mild UWL and abdominal discomfort	None
Male			
High	60	Concerned about UWL, abdominal discomfort, and change in stool color	Family history of colorectal cancer, mild anemia, and thrombocytosis
Low	54	History of mild UWL and abdominal discomfort	None

a UWL: unintended weight loss.

### Study Participants

Recruitment of GPs occurred using various methods: invitation (email or in-person) using the University of Melbourne Department of General Practice and Primary Care contacts; dissemination of study information through advisory groups (eg, Future Health Today Advisory Group) and groups associated with the University (VicREN, a practice-based primary care research network managed by the University of Melbourne and the Primary Care Collaborative Cancer Clinical Trials group); and GP groups on social media platforms (eg, WhatsApp). The recruitment messaging and materials included information about the simulation study, the location of the study, the time required to participate, and reimbursement. Participants were recruited using purposive sampling, with the aim of recruiting a diverse sample in terms of experience, gender, and age. Inclusion criteria applied the following: registered GP, familiar with either Best Practice or Medical Director EMR, who was able to attend the University of Melbourne SimLab for up to 1 hour.

Lived experience community advocates with a history of a cancer diagnosis (henceforth “community advocates” or “CAs”) were recruited via the Department of General Practice and Primary Care newsletter, word of mouth via friends and family, and through organization newsletters such as the Pancare Foundation, Cancer Voices Australia, and the Victorian Comprehensive Cancer Centre. The recruitment messaging and materials included information about the study, the time required to participate, and reimbursement amount. Similar to GP recruitment, purposive sampling was used. CAs were eligible for inclusion if they were over 40, had experience as a general practice patient, and presented with a nonspecific symptom of cancer before being diagnosed.

Interested participants (GPs and patients) were sent further details of the study using a Plain Language Statement. Informed consent was collected from all participants.

### Data Collection

#### Simulated Consultations

Before the simulated consultations began, GPs were introduced to the space and given a brief overview of the tool. GPs had limited information about the FHT module before participating. They were aware that the study evaluated a module for chronic disease but were unaware it was specific to cancer or that it would involve recommendations for UWL. During recruitment, GPs were told that they may be asked to participate in up to two consultations with different patient scenarios (the number of simulated consultations was confirmed on the day of the simulation), but GPs were unaware that two consultations were designed to simulate two different patient risk profiles (one high-risk and one low-risk).

Researchers directed the GP to the appropriate patient record before starting the simulation. GPs were advised to review the patient record (if this was standard practice for them) and call the patient in when ready. The Digital Health Validitron SimLab has a general practice “waiting room,” so the GP and patient actor did not meet before the simulated consultation. GPs were advised to keep the consultation to 10‐15 minutes, in line with the usual duration of GP consultations in Australia. The simulation was filmed, audio recorded, screen recorded (ie, recording the GP’s mouse/cursor movement and clicks when using the computer), and observed by the researchers through a 1-way mirror. Each GP enacted two patient scenarios, one lower and one higher risk, determined by the gender of the patient actor allocated to their session.

#### GP and Actor Interviews

Directly after the two simulated consultations, interviews were conducted with GPs (JMG) and actors (SC). The GP interviews focused on the acceptability, feasibility, and workflow associated with using the UWL module. The interview guides (Multimedia Appendix 2) were developed using 2 frameworks: Acceptability of Health Care Interventions Framework [22] and the Sociotechnical Model for Evaluation of Digital Interventions Framework [23]. Formal data saturation was not deemed necessary to assess study outcomes [24].

Patient actors were asked about their interaction with the GP, the recommended investigations and follow-up plans discussed, and how the tool and its content affect communication (Multimedia Appendix 3).

#### Lived-Experience CA Interviews

From the 20 simulated consultations, two contrasting videos were selected to highlight different GP approaches to incorporating the prompt’s recommendations. One video showed a GP using the FHT prompt to communicate with the patient, while the other depicted a GP who neither used nor acknowledged the prompt during the consultation. These two videos were shown to CAs, followed by a semistructured interview via Zoom conducted by JMG. The interview guide (Multimedia Appendix 4) was developed using the Acceptability of Health Care Interventions Framework [22] and the Sociotechnical Model for Evaluation of Digital Interventions Framework [23].

All interviews (GP, actor, and CAs) were audio recorded and lasted approximately 30‐45 minutes.

### Data Analysis

**Figure 2.**
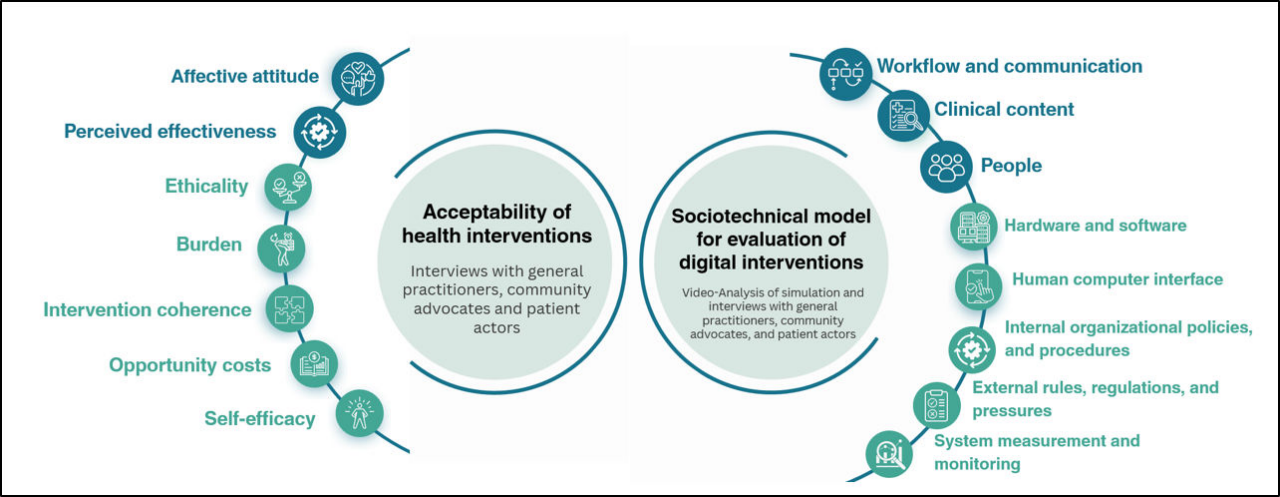
Frameworks used for analysis of interviews and simulated consultations. Adapted from Sekhon’s [22] acceptability of health care interventions and the sociotechnical model for evaluation of digital interventions by Singh and Sittig [23]. Main themes reported are shown in blue.

Otter.Ai was used for the initial transcription, which was then checked by a researcher (SC). Transcripts were then uploaded to NVivo 14, coded, and thematically analyzed [25] independently by JMG and SC. The acceptability of health care interventions underpinned the analysis of GP, CA, and patient actor interviews. Video recordings of the simulated consultations were also analyzed. The video analysis provided additional depth to the overall analysis and was conducted using the Sociotechnical Model (Figure 2). Findings from the interview analysis complemented the themes identified in the video analysis. The two frameworks were used to inform relevant themes, and additional themes were added after the initial review of the data. A subset of participants was asked to provide feedback on the analysis. We report results using the COREQ (Consolidated Criteria for Reporting Qualitative Research) checklist (Checklist 1) [26].

### Ethical Considerations

Ethical approval was granted by the University of Melbourne Human Research Ethics Committee STEMM Three (ethics ID: 28024). Written informed consent was obtained from all study participants before or on the day of the simulation, and all participants were informed of their ability to opt out at any moment. All transcripts and audio recordings were securely stored on an institutional password-protected server without any personal identifiers to maintain participant confidentiality. GPs and CAs were reimbursed for their time (Aus $150 [US $98]). Actors were compensated in accordance with the University of Melbourne’s established salary scale.

### Researchers’ Characteristics and Reflexivity

Two researchers were directly involved in data collection (simulation and interviews) and analysis (JMG and SC). JMG is the lead investigator for this study and was assisted by SC. JMG is a female GP and PhD candidate and midcareer researcher at the Department of General Practice and Primary Care, University of Melbourne. SC is a female early career researcher and postdoctoral fellow in the Department of General Practice and Primary Care. Both researchers have experience in qualitative research, in conducting interviews, and in the development of clinical decision support tools.

## Results

### Simulated Consultations

We conducted a total of 20 simulated consultations involving 10 GPs. Each GP participated in 2 scenarios: 1 high-risk scenario and 1 low-risk scenario. Further, 7 GPs tested the CDSS with the male scenario, and 3 GPs tested it with the female scenario. The patient’s gender in each scenario was determined by the availability of male or female actors on the day of simulation. All GPs were interviewed following the simulations; 2 actors performed the clinical scenarios, and they were interviewed after each GP finished their two clinical interactions (10 interviews with 2 actors). Additionally, 6 CAs were interviewed following their observation of 2 exemplar simulation videos.

GP demographics are summarized in Table 2. All GPs interviewed worked in metropolitan areas of Victoria. There was an even distribution of gender in the sample. Half of the GPs were between 30 and 39 years of age and had <5 years in practice. The age of participating GPs ranged from 31 to 63 years. Interview length ranged from 21 to 32 minutes.

Characteristics of CAs are detailed in Table 3. Half of the CAs were female, and half were between 60 and 69 years of age, with ages ranging from 48 to 71 years. Their year of cancer diagnosis ranged from 2011 to 2018, and they all had consulted their GP with a nonspecific symptom prior to diagnosis, although not necessarily UWL. Interview length ranged from 27 to 57 minutes.

The two actors each played a patient scenario of a middle-aged and older adult. Both patient actors had personal experience as general practice patients. Interviews with the actors were designed to be shorter, with interview length ranging from 5 to 9 minutes.

Characteristics of all participants can be found in Tables2  3.

Consequently, the total number of reported symptoms exceeds the number of CAs interviewed.

**Table 2.** Characteristics of GPs^a^ who participated in the simulated consultations and qualitative interviews.

GP characteristic	GPs (n=10), n
Age (y)	
30‐39	5
40‐49	2
60+	3
Gender	
Male	5
Female	5
Years of experience	
1‐5	5
6‐20	2
>20	3
Practice setting	
Urban	10

a GP: general practitioner.

**Table 3.** Characteristics of community advocates who participated in qualitative interviews.

Community advocate characteristic	Community advocates (n=6)
Age (y)	
40‐49	1
50‐59	1
60+	4
Gender	
Male	3
Female	3
Education	
Finished high school	2
Postgraduate studies	4
Unspecific symptoms experienced prior to their cancer diagnosis^a^	
Urinary symptoms	1
Chest pain	1
Sore throat	2
Muscle sore	1
Lower back pain	1
Persistent flu-like symptoms	1
Fatigue	1
Abdominal pain	1
Change of bowel habits	1

a The number of community advocates experiencing each symptom is reported. Several community advocates reported experiencing multiple symptoms prior to their diagnosis.

### GP and CA Interviews

We present the results for the two main themes in the theoretical framework of acceptability: affective attitude and perceived effectiveness.

#### Affective Attitude

There was a positive attitude toward the UWL module in general, although the reasons for perceived value differed by age and experience. Younger, less experienced GPs tended to value it because it aligned with the way they work (eg, checking guidelines, taking extra time). More experienced GPs valued it primarily as a reminder system.

*Particularly as a younger GP, who looks a lot of things up as I go, I always have eTG up (Therapeutic guidelines), I have health pathways up, I’ll have lots of clinical guidelines up....But that’s what this does for you, it sort of keeps you up to date, saves you time in that it gives you the key information quickly that you would be looking for.*

[GP10]

Prompt fatigue was seen as a barrier for long-term use by some GPs. Half of the GPs felt compelled to follow the recommendation or, when asked directly, questioned the ethical and legal implications of not following them.  

*Yeah, it makes me feel like it’s a must.*

[GP2]

*The only issue I have would be, if I choose to ignore it, and then someone gets diagnosed with cancer, and then I go for a malpractice suit.*

[GP4]

CAs also thought that a module to identify people at risk of undiagnosed cancer had value as a good reminder system. They reflected on how busy GPs can be and felt it could also be used as reassurance to the patients that their GP was considering all diagnostic possibilities. One CA, while acknowledging its usefulness, expressed their concern for the need for tools like this; their perception was that GPs should be able to conduct their clinical practice without any external help.

*The only thing that concerns me is that the doctor shouldn’t need this. I think a competent doctor should know that this patient is presenting with an abnormality, there’s something wrong.*

[CA3]

#### Perceived Effectiveness

Some GPs reported that the CDSS positively impacted their consultation and thought process. They reported that the prompt helped them prioritize investigations and consider differential diagnoses. They also thought it would be a useful tool for safety-netting and ensuring patients are followed up until diagnostic resolution. All GPs found the tool useful as a reminder system for their clinical train of thought but were clear the tool did not replace their clinical acumen.

*It definitely impacted on my thought process approach to the consultation. I do always like to start off with allowing the patient to talk about why they’re here and what’s their concerns, before getting too heavily into more interrogation. But there was a definite need for interrogation based on the prompt, you know, looking at those major, possible causes of unexplained weight loss, both organic and psychological and psychosocial factors as well. So, it helped to structure that.*

[GP6]

However, most GPs, while acknowledging the potential benefits, did not think the tool would change or impact their own clinical practice, often considering that it would be useful for other GPs.

*I personally, probably wouldn’t look at it much. But I think it would be really good, something like this, for people who are just finished their training or sort of registrars and that sort of thing.*

[GP9]

Two GPs reported concerns about EMR data, and the accuracy or sensitivity of the module acknowledging data recording in everyday practice might not be complete enough for this kind of technology. Most GPs reported concerns about trying to explain a very unlikely cancer diagnosis.

*Because, sadly, the notes don’t always accurately reflect the situation with the patient. They’re not always updated. And so, if they’re pulling things out of here, they may not be correct.*

[GP5]

CAs thought it would be most useful to use patients who are new to the clinic or do not have an established relationship with their GP, as they would remind them of possible risk factors and could enable effective communication. CAs and SPs commended the tool’s ability to enhance follow-up care and patient guidance, viewing these features as significant improvements to the consultation process

*With the pop up, what I thought immediately was, he’s not going to miss anything. You know, he can be stressed himself, he could be busy. You know, he might have just had to save someone’s life for all I know. And next minute he’s in here, dealing with me. And you know, he’s human. What I found, what I felt, was when the pop up came up, I was like, he’s not going to miss anything.*

[SP4]

*I think it’s a great idea. I really do. I think it’s a good way to hopefully get a diagnosis sooner, like, without any delay to sort of get right to it. And to not waste time doing this step when you’re really better off going straight to this step here.*

[CA5]

One CA suggested the tool would be especially useful in rural areas, given access to clinical services can be scarce. They also suggested developing multilingual versions to accommodate diverse populations.

### Simulated Consultations and Video Analysis

#### Simulated Consultation Characteristics and Theoretical Framework

The average (range) simulated consultation between GPs and patients lasted 9 minutes and 14 seconds (6 min 30 s to 13 min 23 s). GPs spent an additional average (range) of 1 minute 30 seconds (0-4 min) looking at the recommendations and other relevant information in the EMR (pathology tests and previous visits) before calling the SP in.

We analyzed the interactions between the GP, the tool, and the SP, underpinned by the dimensions of the Sociotechnical Model for Digital Health Interventions. We then identified facilitators and barriers to the use and implementation of the CDSS within each theme. Here we describe the main themes from what was observed in the simulated consultation, complementing these findings with the perceptions of GPs, CAs, and SPs from the interview analysis, where relevant.

#### Workflow and Communication

In the simulated consultations, all GPs reviewed the EMR and CDSS recommendations for a few minutes before calling the patient in. This aligned with what was reported in the interviews, where GPs thought the recommendations were feasible to implement into their workflow. They were unobstructive and easy to read, and most said that they would review the recommendations before the patient came for the consultation in real life. It is important to note that most GPs did not review all features of the prompt (eg, reason for recommendation accessed via hovering over text, links to tailored resources accessed via clickable menu items).

In observing the consultation, half of the GPs acknowledged the prompt with the SP. There were two distinct ways in which this was done during the consultation, in regard to communication: (1) as an aid to involve the patient in the decision-making process and (2) as a reminder or checklist for the doctor to use; some doctors though chose not to discuss the prompt at all in the consultation. These are described further in Table 4.

**Table 4.** Results from the simulation study: how the tool was used when communicating with the patient.

Theme and description	Quote
Aid to involve patients in decision-making	
GPs^a^ integrated the prompt into patient consultations in various ways, often involving patients in decision-making. Some used it to explain their reasoning for follow-up investigations, openly discussing the recommendation with patients. While all GPs adhered to certain aspects of the recommendation, some also explained why they might deviate from it in specific instances.	“I have a little pop-up here, I don’t know if you’ve noticed but it says…doing an FOBT…look, I don’t think a poo test at this point in time is going to add much more...” (GP4, consultation 2 [6 min 49 s]) “Now if you don’t mind, I am just going to have a quick look at my screen, because I have a special thing that tells me…my computer can give me some prompts that actually helps me with your symptoms so that is quite handy.” (GP9, consultation 1 [5 min 10 s])
Some GPs utilized the prompt to broach sensitive topics with new patients, such as mood disorders or potential cancer diagnoses, which might otherwise be challenging to address in an initial consultation.	“Now, I have just received this alert, and I am just going to have a look at what it says. Ok, so this is just prompting me to, as we mentioned, consider testing for your thyroid function as a possible cause, but then also mood disorders, which we started to talk about. Perhaps what I can also add is a screening questionnaire for mental health conditions....” (GP2, consultation 1 [9 min]). “And the depression? Yeah, I went through that. I think that was, it’s a useful reminder to have there” (GP5)
GP reminder and checklist	
Some GPs used the prompt as a checklist for follow-up investigations, often without detailed explanations to patients. In these cases, they focused on addressing key aspects of the recommendation, such as potential diagnoses and suggested tests.	“There is little thing on the screen…(actor asks “does that help you does it”)…it sort of aids the GP at the desktop, just a sort of little prompt.” (GP8, consultation 1 [5 min 25 s]) “I’ll have a little look at a reference list here” while discussing list of investigations. (GP6, consultation 1 [10 min 50 s])
When GPs used the prompt as a checklist without explaining it to patients, both patient actors and community advocates observed a communication gap. Although most GPs did not perceive the tool as disruptive, some concerns arose about its potential to hinder doctor–patient communication.	“I think that there’s room for it to interrupt workflow and your interaction with the patient in a negative way, depending on how you use it.” (GP2) “Yeah it did have an impact on the therapeutic relationship I suppose that there was a dis – that there was a barrier between us.” (SP^b^ 1)
After delivering the recommendation, some GPs consulted the tool to check if there was something missing. This review often prompted additional questions about risk factors or led to the inclusion of further tests, enhancing the thoroughness of the consultation.	“It didn’t bring up any diagnoses that I hopefully wouldn’t have discussed anyway. Oh, except for maybe asking the questions about lung cancer. Yeah, about smoking.” (GP10) At the end of the consultation, GP looks at the screen, reaches for the mouse and says: “Actually, I should have checked. Are you up to date with your cancer screening?” The patient says she is up to date. (GP2, consultation 1 [7 min 5 s])
Not addressed during consultation	
Half of the GPs opted not to discuss the CDSS^c^ tool during consultations, despite considering its recommendations. Reasons varied: some felt confident explaining investigations without referencing the tool, others were cautious about prematurely mentioning cancer, and one GP feared that acknowledging the tool might create distance in the patient interaction.	“I didn’t refer to it as saying, ’oh, this program has recommended I do this.’ Because I think I would only do that if I was sort of trying to distance myself from the recommendation. None of these recommendations are surprising to me, or, you know, difficult to explain. I think they’re all quite common sense. So I, I just use that as a tool, but I’m not saying you know, ’FHT suggests we do this, so we’re going to do this’....” (GP7)

a GP: general practitioner.

b SP: simulated patient.

c CDSS: clinical decision support system.

The use of the tool and the themes around communication was evaluated by both the CAs and SPs. Irrespective of whether the GPs actively discussed their use of the tool or not, both CAs and SPs appreciated GPs who included them in the conversation.

For the CAs, their relationship with their GP and how that shaped their experiences was a core theme throughout the interviews. There were different preferences for communication. Some patients said they would like to be brought into the discussion around use of the tool and would like it explained, while others did not care as long as the GP sounded sure of what the next steps would be.

*There wouldn’t be any particular reason why you would show the patient that pop up, you know, if we calling it that the pop up on the screen, but rather, I would be quite comfortable if the GP said to me in a fairly neutral way “I’m just, you know, I’m being reminded here, because of your presentation that I should check out the following. So that’s what I’m going to do, just to make sure we can eliminate it.” So being told what the person is looking at on the screen also meant you’re being included.*

[CA6]

There were a variety of communication strategies used by GPs throughout the simulated consultations. CAs and patient actors noted how the system was used and/or communicated was very individual. There were instances of both effective and less effective communication as discussed by the patient actors and preferences for how they would like the information delivered, highlighting that there is no one size fits all. The tool could become a facilitator or a barrier to communication depending on its use.

*I don’t think would really matter a great deal whether or not the doctor mentioned the prompt, you know. As long as it’s there to remind the doctor.*

[CA4]

*It all depends on their bedside manner; his bedside manner is really good. I’ve done some simulation with the students over here, and they are nowhere near as good as him, because he’s had years of practice.*

[SP2]

*It’s how they interact with a patient, which is obviously very important.*

[CA1]

#### Clinical Content

GPs thought the wording of the recommendations ensured clinical autonomy (eg, the use of language such as “consider,” “recommended,” instead of more directive words). They also reported that information was well organized and easy to follow. In general, content was considered appropriate and clinically relevant albeit very nonspecific. GPs said they liked that it was symptom-based rather than diagnosis-based and that it included several symptoms at the same time, reflecting real life.

Most GPs’ actions in the consultations were concordant with the tool’s recommendations, suggesting standard pathology testing in primary care to rule out the most common causes of unexpected weight loss by age (eg, thyroid disease, full blood count, general biochemistry, kidney, pancreas, and liver function).

*What is written in there all seems really relevant to me. So, I like that it combined a number of things like, not just the unexpected weight loss, but the patient’s, the patient’s gender, and age to then sort of help stratify risk and saying that, you know, in a patient with unexpected weight loss, of this age, this gender, the things to think about.*

[GP7]

Some tests were considered and discarded after clinical judgment. CA-125 for ovarian cancer was not ordered by any of the GPs in this study despite it being recommended. Four GPs opted against using the recommended immunochemical fecal occult blood test in symptomatic patients, instead favoring more direct diagnostic procedures such as colonoscopy.

*Like you have FOBT testing two years ago, you’re out of date, doing another one now is not really appropriate, because not really screening you anymore.*

[GP5]

For low-risk patients, some GPs favored a phased approach, initially ordering blood tests and deferring imaging to subsequent consultations based on initial results. These GPs expressed concerns about overtesting, citing potential financial burdens and unnecessary anxiety associated with comprehensive investigation. In contrast, for high-risk patients, all GPs recommended more advanced diagnostic procedures, such as endoscopic examinations or computed tomography scans, reflecting a more aggressive investigative strategy.

*The investigations are, you know, also feasible, but I do think as well, system wide issues that probably are unrelated is access to things like these, like you can request all these tests, but whether a patient is going to go and do them and, you know, do they have access to private health insurance to get their private scopes?*

[GP2]

CAs did not care much about the content of the prompt, as long as it was evidence based, and their GP trusted the information.

*I derive a lot of comfort in knowing that it is not random, there is some sort of process, there’s some sort of procedure and it is, you know, it is evidence based, and it is also because of, you know, what I said and what I’m presenting with*

[CA2]

Notably, only a minority of GPs delved into the additional resources provided within the tool, which contained supplementary information. Most reported that the tool itself was sufficiently clear, negating the need for further exploration.

#### People

For both GPs and CAs, it mattered who was using the module. As discussed earlier, differences in years of experience influenced the GPs’ perception of the tool, how they would use it, and what parts they liked. Younger GPs took more time looking at guidelines and considering the recommendations. They mentioned they need to know where the information is coming from to be able to trust it (eg, guidelines sponsored by the Royal Australian College of General Practitioners).

More experienced GPs were observed to be comfortable in the consultation and simulation scenario. Even though they looked at the screen and keyboard when writing, they alternated between the patient and the screen, focusing their attention mostly on the patient. They also felt more comfortable using the tool as an aid to communicate decision-making. Of the five GPs that used and discussed the tool with the patient, four had more than 5 years’ experience as a GP. So, even though younger GPs appreciated the tool as a reminder for their thought process, more experienced GPs were able to use it as a communication tool as well as apply its clinical content.

*We’ve got a nice little pop-up; we have things that listen in the background. And it is saying yes you do have weight loss, and they are worried about things like thyroid function, diabetes and depression. I mean mood wise, how have you been?*

[GP4 with >20 years’ experience]

*I mean, I still interpret. Like here, I still interpret what it’s saying, from my own personal experience and the person in front of me. So, it’s, it’s not telling me what to do. It’s given me advice about what could be done.*

[GP4 with >20 years’ experience]

*Yeah, I think it’s a good idea. I do think, particularly at the early stage of my career now, I probably over investigate, compared to more experienced GPs.*

[GP10 with <5 years’ experience]

As mentioned before, the CA’s and actors’ perceptions on how the tool was used seemed very dependent on the GP’s communications skills—how the tool made them think or feel was directly related to how the GPs relayed the information or used the tool. 

*If I look at it, one was actually using the tool. I hope I get this right in my expression. The tool was driving the session -the second one. First one, the tool was a tool to use, and the doctor was driving the session.*

[CA3]

## Discussion

### Acceptability and Use of UWL CDSS

We tested a CDSS to identify patients with UWL at risk of undiagnosed cancer in a simulated environment. The tool was generally deemed acceptable, particularly as a reminder of differential diagnoses and recommended investigations. However, in line with existing literature, concerns about overreferral and overtesting, as well as reluctance to discuss a potential cancer diagnosis, emerged as potential barriers [27].

The clinical content provided was considered adequate. GPs selectively utilized the information, according to their clinical reasoning influenced by internal (personal experience), external (cost and accessibility), and relationship factors (use of a stepwise approach, deciding to approach a cancer, and mental health discussion later), similar to factors described in international literature [28]. Australian cancer clinical guidelines recommend immunochemical fecal occult blood test as a screening tool for asymptomatic individuals in the general population and also in some symptomatic patients [29,30]. Recent research indicates its effectiveness as a rule-out test for colorectal cancer in patients presenting with gastrointestinal and nonspecific symptoms [31]. There was variable awareness of these recommendations by the GPs, with 6 out of 10 GPs recommending it alongside other imaging for their high-risk patients. CDSSs may be used as facilitators to behavior change, encouraging GPs to adopt new approaches in their diagnostic process. The majority of GPs did not access the tool’s embedded resources, which offered additional context and explanation of the recommendations. This could be explained by the additional cognitive load required to navigate and access less-readily available resources. However, it may also indicate a strong trust in the tool’s core content among participating physicians as confirmed in the interviews. Such trust is noteworthy, as overcoming skepticism toward CDSSs has been identified in literature as a major barrier to their adoption [11].

GPs utilized the tool in diverse ways: incorporating it into their own decision-making process without sharing it with the patient, as a reminder of differential diagnosis and suggested follow-up and to aid communication. Recent evidence showed the potential of a CDSS to improve patient–provider communication in cancer patients [32]. Notably, our CDSS showed the potential to either enhance or impede patient–clinician communication, depending on its application, how GPs integrated it into the consultation, and the specific context of use.

Health care delivery has increasingly become “digitalized”. The use of technology has become embedded in health care systems in most high-income countries, and it continues to expand worldwide. Technology can help clinicians to be more efficient and improve patient safety [33].

Multiple guidelines for digital intervention development have been published by international agencies and government bodies, such as World Health Organization [34], National Institute for Health and Care Excellence [35], the Food and Drug Administration [36], and in the academic literature. In 2020, Mathews et al [37] published the digital health score card, where authors propose that digital health solutions should always be validated from a technical, clinical, and system standpoint [37]. Even with these guidelines, there is not one single framework or process required or agreed upon to test digital interventions in similar real-life scenarios before releasing them to the public [38].

Simulation techniques have emerged as a promising approach to bridge the gap between the development and evaluation of digital health solutions before their implementation [38]. Despite growing interest and well-established methods, simulations for digital interventions prior to real-world deployment are not the norm, and this may impact not only their safety but their potential for implementation, resulting in most tools not being adopted into the routine health care system [39].

Barriers to incorporating new software into health care systems can include concerns about potential impacts on patient care quality, apprehensions regarding data privacy and protection, organizational capacity to manage nonmedical tasks, and the ever-evolving landscape of health care delivery [16].

Our simulation provided a controlled environment to test a CDSS designed to identify individuals with symptoms potentially indicative of undiagnosed cancer. By simulating diverse and realistic clinical scenarios, it allowed the system to be rigorously evaluated without risk to actual patients. It also allowed evaluation to occur in a timely manner, rather than waiting for a real-world “needle in a haystack” identification of low prevalence conditions and symptoms. This approach also offered valuable insights into the barriers and facilitators of implementing a tool for early cancer detection [32].

Notably, the simulation served two key functions. First, it directly modeled and observed practical issues, such as usability and workflow integration. Second, it acted as a “priming” tool, enabling practitioners to provide detailed, informed feedback on the CDSS functionality and recommendations. Most importantly, it highlighted that adequate use and implementation need to consider the “human factor.”

### Strengths and Limitations

A key strength of this study lies in its multimodal analysis approach, combining video recordings of simulations with in-depth interviews with both participants in the consultation and real-live observing CAs. This methodology provided a comprehensive and nuanced understanding of the subject matter, capturing both observable behaviors and subjective experiences. Furthermore, the inclusion of both clinical and lived-experience perspectives ensured a well-rounded analysis, balancing professional insights with authentic patient views. This diverse approach not only enhanced the study’s rigor but also allowed for a comprehensive evaluation that bridges the gap between theoretical design and practical application in health care settings. The additional information gleaned from patients and CAs on how this type of technology and system support can enhance the patient experience in the inclusion of their health management adds an additional perspective to prior research focusing mainly on systems and technological evaluation [37].

All participating GPs were from metropolitan areas. This limitation was primarily due to the logistical requirement for GPs to travel to the simulation laboratory. Additionally, while simulations provide valuable insights, they inherently lack the full complexity and unpredictability of real-world clinical encounters and the pressures of a busy clinical day. This artificial setting may not fully capture the nuances of actual patient interactions, time pressures, or diverse clinical scenarios that GPs encounter in their daily practice, and GPs might act differently than in their regular day-to-day practice when knowing they are being observed.

While most GPs were not previously known to the researchers, and we did not provide information regarding personal goals or reasons for doing the research, two GPs had participated in previous studies and had established working relationships with one or both of the researchers. We acknowledge this may lead to social desirability bias, which we tried to decrease by reassuring them of our intention to assess real implementation, asking questions that could elicit both positive and negative responses toward the tool.

### Conclusions

We developed and evaluated a CDSS designed to identify patients with UWL who may be at risk of undiagnosed cancer. The controlled simulation environment provided a safe setting to assess implementation barriers and facilitators while observing the tool in use. Clinicians found the recommendations to be clear and unobtrusive; however, concerns were raised about potential overtesting in patients with a low risk of cancer. The tool was utilized by clinicians in 3 primary ways: to inform their clinical decision-making process, as a reminder or checklist of best practices, and as an aid in communicating potential follow-up options with patients. Both patients and GPs believed the tool could either enhance or impede communication, depending on its integration into consultations, underscoring the potential need for communication-focused training in the use of these technologies. This study’s findings will inform future studies implementing CDSSs for identifying patients with nonspecific symptoms who may be at risk of cancer, potentially improving early detection and patient outcomes.

## Supplementary material

10.2196/79208 Multimedia Appendix 1 Patient scenarios.

10.2196/79208 Multimedia Appendix 2 General practitioner script.

10.2196/79208 Multimedia Appendix 3 Actors’ script.

10.2196/79208 Multimedia Appendix 4 Community advocate script.

10.2196/79208 Checklist 1 COREQ checklist.

## Abbreviations

CA: community advocate
CDSS: clinical decision support system
COREQ: Consolidated Criteria for Reporting Qualitative Research
EMR: electronic medical record
FHT: Future Health Today
GP: general practitioner
SP: simulated patient
UWL: unintended weight loss
