# Supplementary material for: Evaluating a Clinical Decision Support Tool for Cancer Risk Assessment in Primary Care: Simulation Study of Unintended Weight Loss
Source: JMIR Form Res. 2025 Dec 10;9:e79208. doi: 10.2196/79208 (PMC12694943; doi:10.2196/79208)
Supplement: Multimedia Appendix 1 [file formative-v9-e79208-s001.docx]

Supplementary File 1.0 Patient scenarios

**Patient one: Algorithm 6.b-e and 6.2 (Male, low risk)**

Recommendations with most common differential diagnosis and to ask for symptoms

1. Unexpected weight loss detected in a previous visit: In younger men and women (aged <60 years), prioritise screening for depression and testing for thyroid function and diabetes.
2. Unexpected weight loss detected: Consider further relevant history and investigations. Recommend physical examination including new weight measurement.

**Patient Information**

Name: John Smith

Age: 54

Gender: Male

Occupation: Office Manager

Medical History: No significant medical history

**Chief complaint**

Concerns about abdominal discomfort. He refers to two weeks of bloating and a bit of constipation.

He does not have any other symptoms presently.

*(He consulted 8 weeks ago because he thought he was losing weight.*

*He had a decreased appetite but was not feeling fatigued or had a low mood.*

*His physical examination was normal, and he was asked to come back in a month for another weight measurement, but he forgot.*

*His last weight was 2 kilos more than the actor's weight.)*

**Review of Systems**

Constitutional: Weight loss, fatigue

Gastrointestinal: Abdominal discomfort, change in stool color

Respiratory: No cough, shortness of breath, or chest pain

Cardiovascular: Hypertension, no palpitations or chest pain

Hematologic: No easy bruising or bleeding

Endocrine: No heat or cold intolerance, no excessive thirst or hunger

Neurologic: No headaches, dizziness, or neurological symptoms

**Social history**

John is married and lives with his wife and two teenage children. He reports a balanced diet, no exercise, and no history of substance abuse. He drinks alcohol occasionally on social occasions but does not smoke. He refers to no big stressors and his mood is not depressed.

**Family history**

Dad with diabetes

Mom hypertension

No cancer

**Physical examination:** Normal

**Patient two: Algorithm 6.3 (Male, high risk)**

Recommendations to follow up at increased risk of cancer

1. Unexpected weight loss and abnormal test results. Consider increased risk of cancer as differential diagnosis and investigations such as iFOBT, CXR, upper/lower endoscopy, abdominal CT scan and CA_125 (if female).

**Patient Information**

Name: Peter Brooks

Age: 60

Gender: Male

Occupation: Accountant

Medical History: John has a history of hypertension for which he takes medication. He is a non-smoker and consumes alcohol occasionally. He has not had any significant medical issues in the past.

**Chief Complaint**

Peter presents to your general practice with the chief complaint of unexpected weight loss over the past few months. He reports losing approximately 3 kilograms without any intentional changes to his diet or exercise routine. John states that he has been feeling tired and generally unwell during this period.

**History of Present Illness**

Peter explains that weight loss began around three months ago. He initially attributed it to stress from his busy work schedule and didn't pay much attention to it. However, as the weight loss continued and he started to feel fatigued, he became concerned.

He describes the weight loss as unintentional and occurring despite his regular eating habits. Peter denies any specific changes in appetite, dietary restrictions, or excessive physical activity that might explain his weight loss. He also notes that he's had some mild, intermittent abdominal discomfort and a change in the color of his stools to a pale, clay-like appearance.

**Review of Systems**

Constitutional: Weight loss, fatigue

Gastrointestinal: Abdominal discomfort, change in stool color

Respiratory: No cough, shortness of breath, or chest pain

Cardiovascular: Hypertension, no palpitations or chest pain

Hematologic: No easy bruising or bleeding

Endocrine: No heat or cold intolerance, no excessive thirst or hunger

Neurologic: No headaches, dizziness, or neurological symptoms

Musculoskeletal: No joint pain or muscle weakness

**Social History**

Peter is married and lives with his wife and has 3 adult children. He reports a balanced diet, regular exercise (although he feels less energetic recently), and no history of substance abuse. He drinks alcohol occasionally on social occasions.

**Family History**

Peter's father had a history of colorectal cancer diagnosed in his late 60s, and his mother has hypertension.

**Medications**

Hypertension: Lisinopril 10 mg daily

**Lab test results**

2 weeks ago, he consulted for this reason via telehealth with no other symptoms and the GP ordered basic lab. Results show full blood count test that showed mild iron deficiency anaemia and thrombocytosis.

**Physical Examination**

On examination, Peter appears well-nourished but slightly fatigued. His vital signs, including blood pressure, heart rate, and temperature, are within normal limits. Examination of the abdomen reveals mild tenderness in the right upper quadrant without palpable masses. There is no lymphadenopathy or hepatosplenomegaly.

**Patient three: Algorithm 6.b-e (Female, low risk)**

Recommendations with most common differential diagnosis

1. Unexpected weight loss detected in a previous visit: In women aged 60–79, prioritise testing for thyroid function and screening for depression while considering cancer investigation.

**Patient Information**

Name: Sarah Miller

Age: 60

Gender: Female

Occupation: Teacher

Medical History: Sarah has a history of hypertension. She does not smoke and consumes alcohol occasionally.

**Chief Complaint**

Sarah Miller presents to your general practice with the chief complaint of persistent abdominal pain. She describes the pain as a dull ache in her lower abdomen, which has been present for the past two weeks. The pain is intermittent, but it has been gradually worsening, prompting her visit.

**History of Present Illness**

Sarah explains that the abdominal pain started gradually and is not associated with any specific activities or meals. She denies any recent trauma or injury to the abdomen. Sarah mentions that she hasn't experienced any changes in her bowel habits, such as diarrhea or constipation, and denies any urinary symptoms. She has not noticed any blood in her stool or urine.

*(She consulted 8 weeks ago via telehealth because she thought she was told by a friend she looked like she was losing weight. She had not noticed but consulted anyway as she was feeling a bit stressed. No physical examination was performed given telehealth app, and she was asked to come back in a month for weight measurement, but she went to visit family overseas so could not come to her app.)*

**Review of Systems**

Constitutional: Abdominal pain

Gastrointestinal: No nausea, vomiting, or changes in bowel habits

Urinary: No urinary frequency, urgency, or hematuria

Respiratory: No cough, shortness of breath, or chest pain

Cardiovascular: Hypertension, no palpitations or chest pain

Musculoskeletal: Osteoporosis, no joint pain or muscle weakness

**Social History**

Sarah is a teacher and lives with her partner. She has one adult son. She maintains a balanced diet and stays active with daily walks. She does not smoke and consumes alcohol occasionally on social occasions.

**Family History**

There is no significant family history of gastrointestinal disorders or cancers.

**Medications**

Hypertension: Lisinopril 10 mg daily

**Physical Examination**

On examination, Sarah appears comfortable but slightly distressed due to the abdominal pain. Her vital signs, including blood pressure, heart rate, and temperature, are within normal limits. Abdominal examination reveals mild tenderness in the lower abdomen, primarily in the left lower quadrant, without palpable masses or organomegaly. There is no rebound tenderness or guarding. Normal PR

**Patient four: Algorithm 6.3 (Female, high risk)**

Recommendations to follow up at increased risk of cancer

1. Unexpected weight loss and abnormal test results. Consider increased risk of cancer as differential diagnosis and investigations such as iFOBT, CXR, upper/lower endoscopy, abdominal CT scan and CA_125 (if female).

**Patient Information**

Name: Mary Johnson

Age: 65

Gender: Female

Occupation: Retired

Medical History: Mary was diagnosed 6 months ago with type 2 diabetes, and hypertension. She is a non-smoker and does not consume alcohol. She had a hysterectomy 10 years ago due to uterine fibroids.

**Chief Complaint**

Mary Johnson presents to your general practice with a chief complaint of unexpected weight loss over approx. 1-2 months. She reports losing approximately 4 kilograms without any intentional changes to her diet or exercise routine. Mary mentions feeling increasingly fatigued and weak during this period, which has prompted her visit.

**History of Present Illness**

Mary explains that the weight loss started about three months ago. She initially attributed it to stressors in her personal life and didn't seek medical attention right away. However, as the weight loss continued and her energy levels decreased, she became concerned.

She describes the weight loss as unintentional and occurring despite her regular eating habits. Mary denies any specific changes in appetite, dietary restrictions, or excessive physical activity that might explain her weight loss. She hasn't experienced fever, chest pain, or respiratory symptoms.

**Review of Systems**

Constitutional: Weight loss, fatigue

Gastrointestinal: no nausea or vomiting

Respiratory: No cough, shortness of breath, or chest pain

Cardiovascular: Hypertension, no palpitations or chest pain

Hematologic: No easy bruising or bleeding

Endocrine: No heat or cold intolerance, no excessive thirst or hunger

Neurologic: No headaches, dizziness, or neurological symptoms

Musculoskeletal: Osteoarthritis, no joint pain or muscle weakness

**Social History**

Mary is a widow and lives alone. She maintains a well-balanced diet and engages in light exercise like walking. She does not smoke or consume alcohol. She feels a bit lonely and stressed about her current situation.

**Family History**

Mary has a family history of breast cancer (mother) and heart disease (father).

**Medications**

Diabetes: Metformin 1000 mg twice daily

Hypertension: Lisinopril 20 mg daily

**Lab test results**

6 weeks ago, she came in for a review of her chronic conditions and another GP ordered basic bloods. She had a full blood count test that showed mild iron deficiency anaemia and thrombocytosis.

**Physical Examination**

On examination, Mary appears mildly fatigued. Her vital signs, including blood pressure, heart rate, and temperature, are within normal limits. There is no lymphadenopathy or hepatosplenomegaly. Abdominal examination reveals mild tenderness in the epigastric region, but no masses are palpable. Chest examination is unremarkable.
