# Supplementary material for: Evaluating a Clinical Decision Support Tool for Cancer Risk Assessment in Primary Care: Simulation Study of Unintended Weight Loss
Source: JMIR Form Res. 2025 Dec 10;9:e79208. doi: 10.2196/79208 (PMC12694943; doi:10.2196/79208)
Supplement: Multimedia Appendix 2 [file formative-v9-e79208-s002.docx]

Supplementary File 2.0 GP Interview Guide

**Questions about acceptability (technology in general)**

1. Can you tell me in your own words what you understand FHT to be? *(intervention coherence)*
2. Can you tell me what you think about the use of this program? (Affective attitude)
3. What did you like the most about the tool? (affective attitude)
4. What did you like the least about the tool?
5. Do you have any concerns about the use of a program like this for patients in general practice in general practice? (ethicality)

**Questions relating to the UWL recommendations:**

1. What were your thoughts regarding the use of FHT in consultation?

Potential probing questions:

- 1. If you used it: how did the consultation, go? If not, why did you not use it?
  2. How did it affect the workflow? *(Burden, workflow)*
  3. Were you able to explain to the patient the reason for ordering additional tests or investigations? *(communication)*
  4. Would you like additional clinical and/or patient-facing resources to help explain why additional tests are being ordered?

1. What are your initial thoughts regarding the cancer recommendations?

*Potential probing questions:*

- *How clear are they? (Content)*
- *Did you find any of the recommendations surprising?*
- *Did you need more information to understand any of the recommendations?*
- *How confident were you in your ability to use and understand the FHT recommendations? Is there anything that would make you feel more comfortable? (Self-efficacy)*

1. How useful do you find the recommendations?

*Potential probing questions:*

- *In the context of your everyday practice, how useful do you think the recommendations are for identifying people at risk of cancer? (Burden)*
- *How will/have these influence/d your referral thresholds or test ordering?*
- *Have the cancer recommendations influenced/changed your understanding and management of patients at risk of an undiagnosed cancer? (if yes, how? In what ways?)*
- *Have you been able to use/see the links attached to the recommendations for more information? Are they useful?*

1. Do you think the recommendations and recommended actions are feasible and what impact would they/do they have on your workflow?

*Potential probing questions:*

- *How do you see the management of these recommendations fitting into usual practice? Do you see a role for the practice managers or nurses using the dashboard for recall when it comes to the cancer recommendations?*
