# Supplementary material for: Evaluating a Clinical Decision Support Tool for Cancer Risk Assessment in Primary Care: Simulation Study of Unintended Weight Loss
Source: JMIR Form Res. 2025 Dec 10;9:e79208. doi: 10.2196/79208 (PMC12694943; doi:10.2196/79208)
Supplement: Multimedia Appendix 3 [file formative-v9-e79208-s003.docx]

Supplementary File 3.0 Patient Actor Interview Guide

Questions evaluating the module as a health information technology intervention:

1. How clear was the GP explaining the recommendation to you? How appropriate was the content and amount of information discussed by the GP? (Content and communication)
2. How did the use of this technology affect the interaction and communication between you and the GP? Did it distract you? (Human-computer interaction)
3. What would your takeaway from this consultation be (is there an urgency to act? Was it stressful?)
