# Supplementary material for: Evaluating a Clinical Decision Support Tool for Cancer Risk Assessment in Primary Care: Simulation Study of Unintended Weight Loss
Source: JMIR Form Res. 2025 Dec 10;9:e79208. doi: 10.2196/79208 (PMC12694943; doi:10.2196/79208)
Supplement: Multimedia Appendix 4 [file formative-v9-e79208-s004.docx]

Supplementary File 4.0 Community Advocate Interview Guide

**Questions about acceptability**

1. Can you tell me in your own words what you understand FHT to be? (intervention coherence)
2. Can you tell me what you think about the use of this program? (Affective attitude)
3. Do you have any concerns about the use of a program like this for patients in general practice in general practice? (ethicality)
4. How easy do you think it was for the patient to hear the recommendation and need for follow-up? (Burden)

***For people who observed the interaction***

Questions evaluating the module as a health information technology intervention.

1. How clear was the GP explaining the recommendation to the patient? How appropriate was the content and amount of information discussed by the GP? *(Content and communication)*
2. Did you notice the GP interacting with the technology (FHT or the computer generally)? Do you feel this impacted the interaction between GP and patient? In what way? *(Human-computer interaction)*
3. How was the recommendation introduced into the conversation? Did this affect the rapport and communication between the patient and GP? *(Communication)*
4. Do you think this interaction is reflective of how unspecific symptoms are usually addressed/followed-up in general practice? Can you expand on the key differences? *(People)*
5. What would your takeaway from this consultation be (is there an urgency to act? Was it stressful?)
