# Supplementary material for: Evaluating a Clinical Decision Support Tool for Cancer Risk Assessment in Primary Care: Simulation Study of Unintended Weight Loss
Source: JMIR Form Res. 2025 Dec 10;9:e79208. doi: 10.2196/79208 (PMC12694943; doi:10.2196/79208)
Supplement: Checklist 1 [file formative-v9-e79208-s005.pdf]

Supplementary File 5.0

Coreq Checklist.

			Reported?
**Domain 1: Research team and reflexivity**			
Personal Characteristics			
1.	Interviewer/facilitator	Which author/s conducted the interview or focus group?	Page 6, Line 152: “Directly after the two simulated consultations, interviews were conducted with GPs (JMG) and actors (SC)”.
2.	Credentials	What were the researcher's credentials? *E.g. PhD, MD*	Page 9, Line 192 under “Researchers’ characteristics and reflexivity”
3.	Occupation	What was their occupation at the time of the study?	Page 9, Line 192 under “Researchers’ characteristics and reflexivity”
4.	Gender	Was the researcher male or female?	Page 9, Line 192 under “Researchers’ characteristics and reflexivity”
5.	Experience and training	What experience or training did the researcher have?	Page 9, Line 192 under “Researchers’ characteristics and reflexivity”
Relationship with participants			
6.	Relationship established	Was a relationship established prior to study commencement?	Page 22, Line 491: “While most GPs were not previously known to the researchers, and we did not provide information regarding personal goals or reasons for doing the research, two GPs had participated in previous studies and had established working relationships with one or both of the researchers.”
7.	Participant knowledge of the interviewer	What did the participants know about the researcher? e.g. *personal goals, reasons for doing the research*	Page 22, Line 491: We did not provide information regarding personal goals or reasons for doing the research.
8.	Interviewer characteristics	What characteristics were reported about the interviewer/facilitator?	Page 22, Line 491: We did not provide information regarding personal goals or reasons for doing the research.

		e.g. *Bias, assumptions, reasons and interests in the research topic*	
**Domain 2: study design**			
Theoretical framework			
9.	Methodological orientation and Theory	What methodological orientation was stated to underpin the study? *e.g. grounded theory, discourse analysis, ethnography, phenomenology, content analysis*	Page 7, Line 171: "Transcripts were then uploaded to NVivo 14, coded and thematically analysed[25] independently by JMG and SC. The Acceptability of Healthcare Interventions underpinned the analysis of GP, community advocate and patient actor interviews. Video recordings of the simulated consultations were also analysed. The video analysis provided additional depth to the overall analysis and was conducted using the Sociotechnical Model"
Participant selection			
10.	Sampling	How were participants selected? *e.g. purposive, convenience, consecutive, snowball*	Page 5, Line 118: "Participants were recruited using purposive sampling, with the aim of recruiting a diverse sample in terms of experience, gender and age"
11.	Method of approach	How were participants approached? e.*g. face-to-face, telephone, mail, email*	Page 5, Line 111: "Recruitment of GPs occurred using various methods: invitation (email or in-person) using the University of Melbourne Department of General Practice and Primary Care contacts; dissemination of study information through advisory groups (e.g., Future Health Today Advisory Group) and groups associated with the University (VicREN, a practice-based primary care research network managed by the University of Melbourne, and the Primary Care Collaborative Cancer Clinical Trials group); and GP groups on social media platforms (e.g., WhatsApp)"
12.	Sample size	How many participants were in the study?	Page 9, Line 200: "We conducted a total of 20 simulated consultations involving 10 GPs"
13.	Non-participation	How many people refused to participate or dropped out? Reasons?	No participants refused or dropped out, they just did not respond to emails.
Setting			

14.	Setting of data collection	Where was the data collected? e.*g. home, clinic, workplace*	Page 6, Line 152: Directly after the two simulated consultations, interviews were conducted with GPs (JMG) and actors (SC).
15.	Presence of non-participants	Was anyone else present besides the participants and researchers?	No one else was present
16.	Description of sample	What are the important characteristics of the sample? *e.g. demographic data, date*	Page 6, Line 199: Tables 2 and 3
Data collection			
17.	Interview guide	Were questions, prompts, guides provided by the authors? Was it pilot tested?	Yes. Scripts provided in the supplementary materials
18.	Repeat interviews	Were repeat interviews carried out? If yes, how many?	We did not conduct any repeat interviews
19.	Audio/visual recording	Did the research use audio or visual recording to collect the data?	Page 6, Line 148:" The simulation was filmed, audio-recorded, screen-recorded (i.e., recording the GP's mouse/curser movement and clicks when using the computer) and observed by the researchers through a one-way mirror."<br><br>Page 7, Line 169: "All interviews (GP, actor, CAs) were audio recorded and lasted approximately 30-45 minutes."
20.	Field notes	Were field notes made during and/or after the interview or focus group?	No field notes were produced
21.	Duration	What was the duration of the interviews or focus group?	Page 7, Line 169: "All interviews (GP, actor, CAs) were audio recorded and lasted approximately 30-45 minutes."
22.	Data saturation	Was data saturation discussed?	Page 6, Line 156: "Formal data saturation was not deemed necessary to assess study outcomes[24]."

23.	Transcripts returned	Were transcripts returned to participants for comment and/or correction?	The transcripts were not returned.
**Domain 3: analysis and findings**			
Data analysis			
24.	Number of data coders	How many data coders coded the data?	Page 7, Line 171: "Transcripts were then uploaded to NVivo 14, coded and thematically analysed [25] independently by JMG and SC".
25.	Description of the coding tree	Did authors provide a description of the coding tree?	Code tree not provided
26.	Derivation of themes	Were themes identified in advance or derived from the data?	Page 6, Line 177: "The two frameworks were used to inform relevant themes, and additional themes were added after the initial review of the data."
27.	Software	What software, if applicable, was used to manage the data?	Page 7, Line 172: "Transcripts were then uploaded to NVivo 14, coded and thematically analysed."
28.	Participant checking	Did participants provide feedback on the findings?	Page 7, Line 171: "A subset of participants was asked to provide feedback on the analysis."
Reporting			
29.	Quotations presented	Were participant quotations presented to illustrate the themes / findings? Was each quotation identified? e*.g. participant number*	In results: Pages 11 to 20.
30.	Data and findings consistent	Was there consistency between the data presented and the findings?	In results: Pages 11 to 20.
31.	Clarity of major themes	Were major themes clearly presented in the findings?	In results: Pages 11 to 20.

32.	Clarity of minor themes	Is there a description of diverse cases or discussion of minor themes?	In results: Pages 11 to 20.

Tong A, Sainsbury P, Craig J. Consolidated criteria for reporting qualitative research (COREQ): a 32-item checklist for interviews and focus groups. International journal for quality in health care. 2007 Dec 1;19(6):349-57.
